# COVID-19-Induced Encephalitis: A Case Report of a Rare Presentation With a Prolonged Electroencephalogram

**DOI:** 10.7759/cureus.14476

**Published:** 2021-04-13

**Authors:** Mohammed A Miqdad, Saed Enabi, Mohammad Alshurem, Tariq Al-Musawi, Abdullah Alamri

**Affiliations:** 1 Internal Medicine, Dr. Sulaiman Al-Habib Hospital, Khobar, SAU; 2 Internal Medicine, California Institute of Behavioral Neurosciences & Psychology, Fairfield, USA; 3 Neurology, Imam Abdulrahman Bin Faisal University, Khobar, SAU; 4 Intensive Care Unit, Dr. Sulaiman Al-Habib Hospital, Khobar, SAU; 5 Associate professor in medicine, Royal College of Surgeons in Ireland - Medical University of Bahrain, Manama, BHR; 6 Neurology Department, King Fahd Hospital of the University, Imam Abdulrahman Bin Faisal University, Khobar, SAU

**Keywords:** covid 19 encephalitis, corona virus, sars-cov-2, encephalitis, confusion, theta wave, acyclovir

## Abstract

Encephalitis is one of the rare complications of coronavirus disease 2019 (COVID-19) that can be missed and confused with other causes of encephalitis. There was a 36-year-old male known to have glucose-6 phosphate dehydrogenase deficiency, who was brought to the emergency department with fever and confusion of one-week duration. Altered mental status work-up, including cerebrospinal fluid analysis, was done and turned out to be nondiagnostic. Multiple prolonged video-electroencephalographic recordings were done and showed different abnormalities suggestive of encephalitis. The diagnosis of COVID-19-induced encephalitis was made by exclusion of other encephalitis-related etiologies in the presence of a positive COVID-19 polymerase chain reaction (PCR) test, and treatment was initiated accordingly. Over a period of three weeks, the patient showed progressive improvement and was discharged home with regular follow-up in the neurology clinic. Upon follow-up in the clinic, the patient was fully independent but with multiple abnormal electroencephalographic recordings showing generalized encephalopathy with no epileptic discharges.

## Introduction

Coronavirus disease (COVID-19) is an infection caused by severe acute respiratory syndrome coronavirus-2 (SARS-CoV-2). According to the World Health Organization, to date, COVID-19 infected more than 120 million people worldwide and resulted in more than two million deaths. Most people affected by COVID-19 have mild self-limiting upper respiratory symptoms. However, critical complications have been reported, such as acute respiratory distress syndrome, acute heart failure, acute kidney injury, sepsis, and life-threatening metabolic disorders, especially among patients with old age or underlying chronic comorbidities [1]. Moreover, neurological manifestations have also been reported, although rare [1,2]. Common neurological symptoms have been reported, like anosmia and ageusia, which can be present in the absence of other symptoms [3]. Uncommonly, COVID-19 can be manifested with ischemic stroke, meningoencephalitis, intracerebral hemorrhage, acute myelitis, and peripheral nerve disorders, such as Guillain-Barre syndrome (GBS) and Bell's palsy [1,2]. A review study published on July 2, 2020, discussed 901 patients with COVID-19-related neurological manifestations [3]. Encephalopathy has been reported in 56/272 patients (20.6%) and encephalitis in eight patients [3]. 

Encephalitis is an inflammation of the brain tissues, usually caused by an infection or an autoimmune phenomenon [3]. Although encephalitis is considered to be a histopathological diagnosis, clinical diagnosis can be based on cerebrospinal fluid (CSF) pleocytosis, brain image changes, and/or abnormal electroencephalogram (EEG) findings [3]. The pathophysiological process behind COVID-19-related encephalitis is not fully elucidated [4]. It is thought to be related to the immunological response against the virus that may lead to brain tissue inflammation with or without edema; consequently, leading to alteration in the conscious level [4]. Ordinarily, viruses may enter the central nervous system (CNS) by different paths, including bloodstream, olfactory and trigeminal nerves, and lymphatic system [4]. Nevertheless, the precise path by which SARS-CoV-2 enters the CNS has still not been recognized yet [4]. Various evidence showed that SARS-CoV-2 initially invaded peripheral nerve terminals and obtained their access to the CNS through trans-synaptic transfer [4].

## Case presentation

A 36-year-old male known to have glucose-6 phosphate dehydrogenase deficiency was presented with a one-week history of fever followed by cognitive impairment and decreased responsiveness. Before admission, he was screened for COVID-19 due to low-grade fever and contact with a suspected COVID-19 patient, but the result was negative. He was brought by ambulance to the emergency department, where he found to be stuporous but with stable vital signs. Neurological examination showed decreased consciousness level where he was opening his eyes to the pain, verbalizing noncoherent words, and localizing to pain. As a result, the patient was admitted to the intensive care unit. 

**Figure 1:**
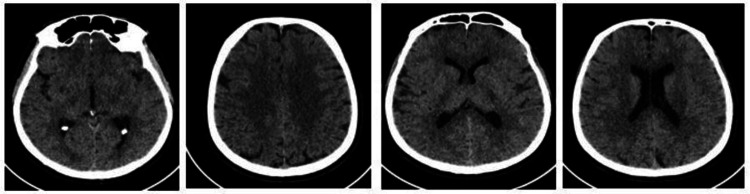
CT non-contrast of the brain. Impression: normal CT brain.

**Figure 2:**
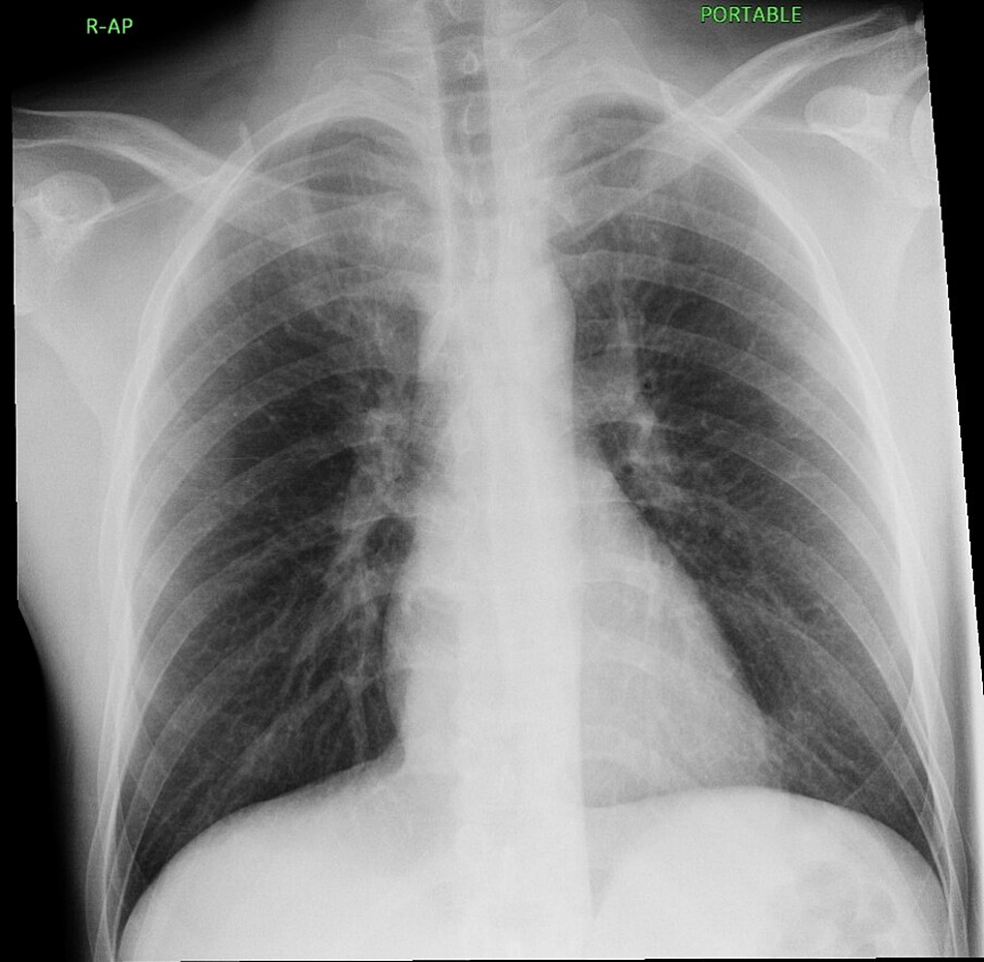
Portable chest x-ray Both lung fields were clear, normal cardiothoracic ratio, normal hilar shadows, and central mediastinum. Both costophrenic angles were clear. The bony cage is intact, and no air under the diaphragm.

Laboratory investigations, including the CSF study and COVID-19 polymerase chain reaction (PCR) swab, were sent (Table 1). The swab of COVID-19 was positive, inflammatory markers, including C-reactive protein (CRP), white blood cells (WBC), D-dimer, and procalcitonin, were high. Blood and urine culture and sensitivity showed no growth, and platelets were adequate. Initial radiological studies, including chest X-ray and non-contrast CT scan of the brain, were unremarkable (Figures 1, 2).

**Table 1:** Laboratory Investigations Hb: hemoglobin; WBC: white blood count; PTT: partial thromboplastin time; INR: international normalized ratio; LDH: lactate dehydrogenase; CRP: C-reactive protein; ALT: alanine aminotransferase; AST: aspartate aminotransferase

Laboratory Investigations	
Hb	11.2 g/dL
WBC	16.10 uL (Neutrophilia, Lymphopenia)
Platelets	170 10^3^/uL
PTT	27.1 seconds
INR	1.07
Creatinine	76.2 umol/L
Urea	4.60 mmol/L
K	3.3 mmol/L
Na	131 mmol/L
LDH	175 mmol/L
Ferritin	883
D-dimer	1.01 mg/L FEU
CRP	71 mg/L
Procalcitonin	1.59 ng/ml
ALT/AST	19/15 IU/L

**Figure 3:**
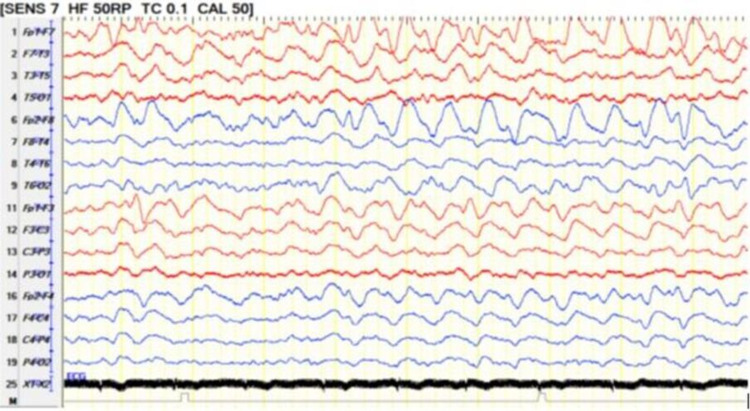
Twenty-four hours EEG 24-hour EEG on bipolar montage showing absence of posterior background activity, there is continuous 2-3 Hz delta slowing generalized maximum in the bifrontal region consistent with moderate diffuse encephalopathy. EEG: electroencephalogram

**Figure 4:**
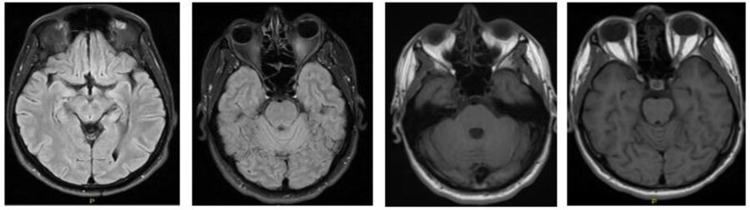
MRI brain upon admission Multiplanar, multi-sequential contrast MRI study of the brain with contrast. Both images showed the following: no evidence of acute infarction of intracranial hemorrhage. No space-occupying lesion. No abnormal focal parenchymal of meningeal enhancement. Normal appearance of the circle of Willis. No evidence of dual venous sinus thrombosis.

**Figure 5:**
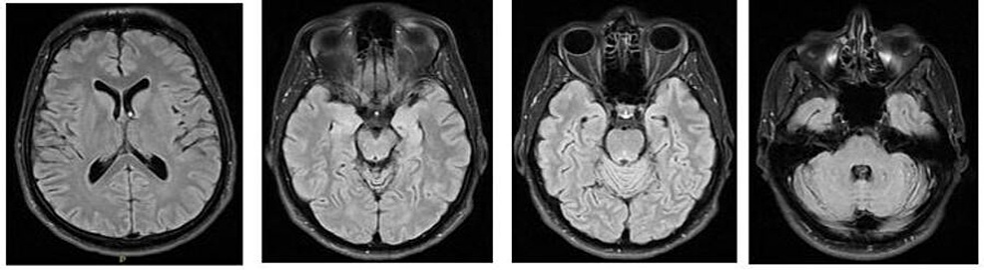
Follow-up brain MRI showing unremarkable study

**Figure 6:**
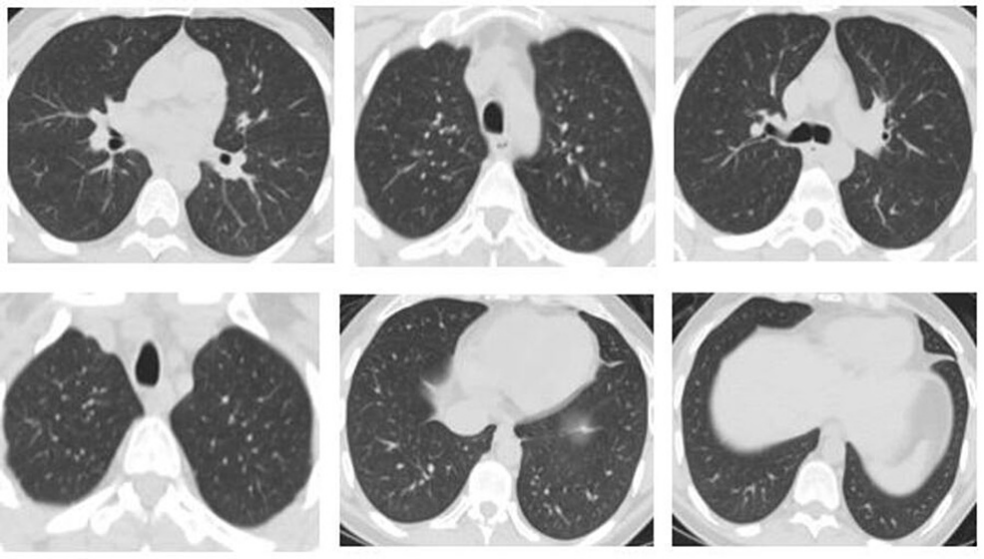
CT scan plain for the chest showed unremarkable findings.

**Figure 7:**
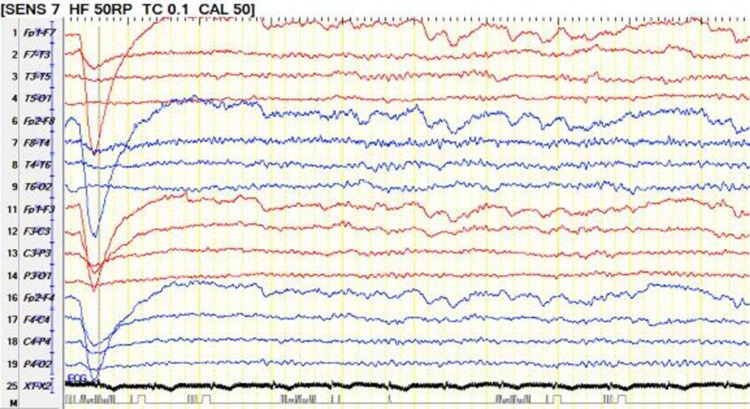
Follow-up video-EEG recording (for 24-hour) showed on bipolar montage showing moderate diffuse encephalopathy with evidence of cerebral dysfunction over the left frontocentral region EEG: electroencephalogram

Empirical triple antimicrobial therapy (ceftriaxone/vancomycin/acyclovir) meningitis dosing was administered in addition to dexamethasone. First prolonged video-EEG monitoring (24-hour recording) showed generalized encephalopathy-2-3 Hz delta waves (Figure 3). CSF study results came back unremarkable for which antibacterial (ceftriaxone/vancomycin) were discontinued (Table 2). An enhanced cranial MRI and CT scan of the chest was unremarkable (Figures 4-6). Over a period of 10 days, the patient started to show a gradual improvement in the conscious level and was transferred to the medical ward. Aciclovir was discontinued later based on a negative herpes simplex virus-polymerase chain reaction (HSV-PCR) result from the CSF sample. A follow-up prolonged video-EEG monitoring (for 24 hours) showed moderate diffuse encephalopathy with evidence of cerebral dysfunction over the left frontocentral region (Figure 7). 

**Table 2:** CSF study HSV-PCR: herpes simplex virus-polymerase chain reaction; WBC: white cell count; RBCs: red blood cells; CSF: cerebrospinal fluid

CSF study	
Appearance	Turbid
Color	Pale red
WBCs	302 cell/uL; Lymphocyte: 85% and Neutrophil: 15%
Glucose	2.59 mg/dL
Protein	832
Culture and sensitivity	No growth
HSV-PCR	Negative
RBCs	3425 cell/uL
Gram stain	Negative

**Figure 8:**
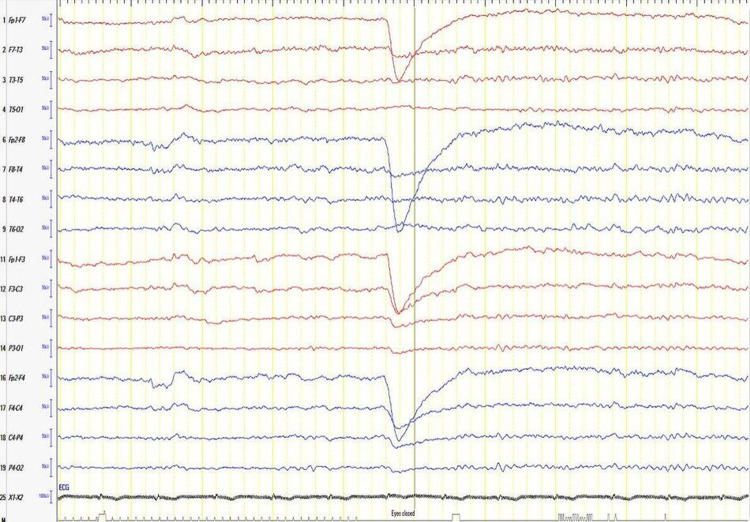
EEG after discharge A routine EEG after discharge showing the posterior background of 9-10 Hz and intermittent polymorphic 2-5 Hz slowing generalized that indicate mild diffuse encephalopathy. EEG: electroencephalogram

Although the patient showed continuous improvement clinically, a second COVID-19 PCR swab turned out to be positive. However, the third COVID-19 swab came back negative, for which he was discharged with regular follow-up in the neurology clinic. Upon follow-up in the neurology clinic, he showed significant neurological improvement with unremarkable laboratory investigations. A routine EEG follow-up showed a normal posterior background of 9-10 Hz, but intermittent polymorphic 2-5 Hz slowing generalized, indicating a mild diffuse encephalopathy (Figure 8). Although a specific test for CSF SARS-CoV-2 was unavailable, the final diagnosis was made on the basis of clinical presentation and supportive laboratory and EEG findings in the absence of other common encephalitis-related etiologies. Up to our knowledge, this is the first case report with multiple prolonged EEG recordings.

## Discussion

COVID-19-induced encephalitis is not a well-known or well-reported complication of the COVID-19 [5]. Very few cases were reported worldwide, and no guidelines were established to treat COVID-19-induced encephalitis, for which each country follows its own protocols [5]. It is worth mentioning that the exact mechanism of how COVID-19 causes brain damage is not yet clear; however, some hypotheses have been put forward [5]. One of the hypotheses is that the coronavirus has the capacity to bind to angiotensin-converting enzyme 2 (ACE 2) receptors in the brain, which is known to regulate blood pressure and play a role in the blood-brain barrier (BBB) integrity [5]. Damage to those receptors can lead to an elevation in blood pressure and possible disruption of the BBB [5]. Moreover, COVID-19 infection can lead to immunological disturbances, directly affecting the cerebral spheres [6]. 

The first case reported in the literature was from Wuhan, China, about a 24 years old man with no significant past medical history brought to the hospital after being collapsed at home [7]. His initial Glasgow Coma Scale was six, and he also had neck stiffness, high CRP, and elevated WBC; consequently, CT brain was done and showed no evidence of any brain injury [7]. CT-chest showed a small ground glass appearance on both lungs, and lumbar puncture (LP) was performed, showing a positive result of COVID-19, although the nasopharyngeal swab was negative [7]. In the emergency department, he developed multiple seizures and was then intubated on a mechanical ventilator [7]. He was diagnosed with meningitis and viral pneumonia, for which he was started empirically on ceftriaxone, vancomycin, acyclovir, steroid, and levetiracetam for the seizure [7]. MRI was done and showed lateral ventriculitis and encephalitis [7]. After 15 days, the patient was declared dead, and the specimen was taken from his brain, which indicated a positive result for the COVID-19 [7].

Another case report is about a 36-year-old male who has no past medical history presented with a complaint of fever, vomiting, headache, and confusion [2]. Neurological examination was unremarkable, CBC showed mild leukocytosis, and CRP was normal; further, his chest X-ray was unremarkable [2]. COVID-19 swap was taken, which came back positive together with a brain CT that showed right frontal hematoma and subarachnoid hemorrhage [2]. The hematoma was surrounded by edema and causing a midline shift [2]. The aforementioned findings were attributed to viral encephalitis [2]. CT angiogram was done, and it came back negative for arteriovenous (AV) malformation or thrombosis [2]. The patient was transferred to the neuro-intensive care unit (ICU) for supportive management, for which the intracerebral hemorrhage was reabsorbed, and the subdural hematoma was evacuated by Burr hole 12 days later [2]. After stabilizing the patient, a lumbar puncture was done, and it came back positive for the COVID-19 virus [2].

The first case report for a pediatric age group was also from Wuhan, China, an 11 years old boy who was presented with status epileptics, for which four anticonvulsant medications were given to abort it [8]. CSF analysis was consistent with viral encephalitis, and EEG was done showing multiple intermittent delta activities [8]. The patient was treated conservatively and discharged home after two negative swabs for COVID-19 [8].

As mentioned above, our patient presented with unusual symptoms of COVID-19 infection. The patient was managed closely and extensively investigated. Other encephalitis-related infections, including arthropod-borne infection, were excluded based on the clinical scenario, laboratory investigations, and epidemiological distribution in the presence of COVID-19-positive swabs. A specific test to detect COVID-19 in the CSF was unavailable at that time in our hospital. To our knowledge, this is the first case of COVID-19-induced encephalitis with prolonged video-EEG recordings reported in Saudi Arabia.

## Conclusions

COVID-19 can lead to various neurological complications, including central and peripheral diseases. Rarely, COVID-19 can lead to encephalitis that can be easily misdiagnosed. Attention to such a CNS complication in the era of the COVID-19 pandemic can prevent further neurological complications. Prospective multi-center studies investigating such a rare presentation should be conducted.
